# Public Health Academic Alliance for COVID-19 Response: The Role of a National Medical Task Force in Puerto Rico

**DOI:** 10.3390/ijerph17134839

**Published:** 2020-07-05

**Authors:** Marcia Cruz-Correa, Elba C. Díaz-Toro, Jorge L. Falcón, Enid J. García-Rivera, Humberto M. Guiot, Wanda T. Maldonado-Dávila, Karen G. Martínez, William Méndez-Latalladi, Cynthia M. Pérez, Myrna L. Quiñones-Feliciano, Juan Carlos Reyes, Pablo Rodríguez, Jorge Santana-Bagur, Luis C. Torrellas, Dharma Vázquez, Guillermo J. Vázquez, Segundo Rodríguez-Quilichini

**Affiliations:** 1 Department of Internal Medicine, School of Medicine, Medical Sciences Campus, University of Puerto Rico, San Juan 00936, Puerto Rico, USA; marcia.cruz1@upr.edu (M.C.-C.); jorge.santana3@upr.edu (J.S.-B.); 2 Division of Cancer Biology, University of Puerto Rico Comprehensive Cancer Center, San Juan 00936, Puerto Rico, USA; 3 Department of Restorative Sciences, School of Dental Medicine, Medical Sciences Campus, University of Puerto Rico, San Juan 00936, Puerto Rico, USA; elba.diaz@upr.edu; 4 Department of Emergency Medicine, School of Medicine, Medical Sciences Campus, University of Puerto Rico, San Juan 00936, Puerto Rico, USA; jorge.falcon1@upr.edu; 5 Endowed Health Services Research Center, School of Medicine, Medical Sciences Campus, University of Puerto Rico, San Juan 00936, Puerto Rico, USA; enid.garcia3@upr.edu; 6 Department of Microbiology and Medical Zoology, Medical Sciences Campus, University of Puerto Rico, San Juan 00936, Puerto Rico, USA; guillermo.vazquez1@upr.edu; 7 School of Pharmacy, Medical Sciences Campus, University of Puerto Rico, San Juan 00936, Puerto Rico, USA; wanda.maldonado1@upr.edu; 8 Department of Psychiatry, School of Medicine, Medical Sciences Campus, University of Puerto Rico, San Juan 00936, Puerto Rico, USA; 9 Department of Surgery, School of Medicine, Medical Sciences Campus, University of Puerto Rico, San Juan 00936, Puerto Rico, USA; william.mendez1@upr.edu (W.M.-L.); pablororc@gmail.com (P.R.); segundo.rodriguez@upr.edu (S.R.-Q.); 10 Graduate School of Public Health, Medical Sciences Campus, University of Puerto Rico, San Juan 00936, Puerto Rico, USA; cynthia.perez1@upr.edu (C.M.P.); juan.reyes5@upr.edu (J.C.R.); dharma.vazquez@upr.edu (D.V.); 11 Department of Pediatrics, School of Medicine, Medical Sciences Campus, University of Puerto Rico, San Juan 00936, Puerto Rico, USA; myrna.quinonez@upr.edu; 12 Puerto Rico Trauma Center, San Juan 00935, Puerto Rico, USA; 13 Menonita Health System, Aibonito 00705, Puerto Rico, USA; lctorrellasmd@gmail.com

**Keywords:** COVID-19, preparedness, Puerto Rico, Medical Task Force, public health, academic alliance, crisis management

## Abstract

Alliances between the government and academic communities can be a key component of the public health response to an emergency such as the coronavirus disease 2019 (COVID-19) pandemic. The Governor of Puerto Rico designated the Puerto Rico Medical Task Force (MTF) COVID-19 to provide direct guidance and evaluation of the government response to the epidemic in Puerto Rico. Several work groups were formed within the MTF to create protocols and provide evidence-based recommendations on different public health aspects. The collaboration between the academia and the government enhanced the Puerto Rican public health response and contributed to the reduction seen in the contagion curve. Healthcare services and hospitals have not reached their maximum patient care capacity and the death toll has been controlled. Incorporating a national MTF with members of the academia into the government structure was beneficial during the COVID-19 response in Puerto Rico. A similar strategy could serve as a model for other states or territories and countries in similar scenarios.

## 1. Introduction

Puerto Rico faced the difficult task of preparing again for another natural disaster. After the catastrophic 2017 hurricane season left Puerto Rico without its essential services infrastructure, the island had a significant earthquake sequence that started in December 2019. These adversities increased the healthcare disparities among vulnerable populations in many parts of the island [1,2,3].

The first case of coronavirus disease 2019 (COVID-19) was reported in Puerto Rico on 13 March 2020, and two days later, a shelter-in-place order for Puerto Rico was established at a time when the World Health Organization (WHO) transmission pattern in Puerto Rico was still classified as sporadic cases (countries/territories/areas with one or more cases, imported or locally detected) [4]. On 19 March 2020, the Governor of Puerto Rico, Hon. Wanda Vázquez-Garced, appointed the Puerto Rico Medical Task Force (MTF) COVID-19 in order to assist the Puerto Rico Department of Health in establishing an early, effective public health response to this epidemic.

The purpose of the Puerto Rico MTF COVID-19 was to congregate a group of volunteer scientists and physicians who would advise the Governor on the preparation, rapid response, and management of the epidemic within the island. The group would develop evidence-based recommendations that would include an up-to-date literature review about severe acute respiratory syndrome coronavirus 2 (SARS-CoV-2), COVID-19, and strategies to adapt the public health response to the reality of Puerto Rico. The principal objectives of the MTF were as follows: (1) to reduce and stop the transmission of the virus by doing rapid detection of cases and, in turn, to prevent deaths; (2) to provide clinical guidelines on the appropriate medical management of cases, including critically ill patients; and (3) to minimize the impact of the epidemic on the healthcare system, social services, and community in general.

Although the Government of Puerto Rico had the public health response infrastructure within its Department of Health, the Governor appointed the MTF to serve as an independent advisory board. Using the University of Puerto Rico and its academic faculty also allowed the Department of Health to focus on implementation while the MTF reviewed the evidence regarding this novel disease. Puerto Rico, as an unincorporated territory of the United States (U.S.), receives the guidance of the federal government on how to manage public health emergencies. However, given our distinct social, cultural, and geographical characteristics, it was important to adapt these recommendations to the needs of Puerto Rico. The weakened healthcare system and disparities in testing capacity and federal healthcare funding in the island represented enormous challenges, but the collaboration with the academia through the MTF had the potential to provide the expertise needed to advise the Governor on how to quickly integrate the scientific recommendations into a public health response. To this date, the strategies designed and implemented have prevented a collapse in the healthcare system in Puerto Rico during the COVID-19 pandemic and transmission of the virus has remained under control, as compared with other states or territories and countries. This paper summarizes the role of a national MTF during the COVID-19 response in Puerto Rico and describes the achievements of the group in the pandemic control.

## 2. Materials and Methods

### 2.1. MTF Development and Composition

The members of the Puerto Rico MTF COVID-19 were chosen from the University of Puerto Rico (UPR), Medical Sciences Campus (MSC) faculty to represent the different perspectives needed to meet its objectives. Participants included public health specialists and physicians with extensive clinical and research experience. It was clear that not all health sectors could be included within the MTF, so each member was expected to establish collaborations with other health professionals, public and private entities, and professional organizations in order to strengthen the health system’s response to COVID-19.

The UPR is the public university system of Puerto Rico. Its MSC includes the School of Medicine and Biomedical Sciences, the School of Dental Medicine, the School of Pharmacy, the Graduate School of Public Health, the School of Nursing, and the School of Health Professions. The MSC is a leader institution in teaching, research, and clinical services in Puerto Rico and the Caribbean. In addition to the appointed members, there were collaborators who represented other health professions and medical specialties as well as representatives from the major professional organizations of Puerto Rico. The diversity of backgrounds of the MTF members and collaborators correlates with the interprofessional model of healthcare.

### 2.2. MTF Organization and Development of Protocols and Recommendations

The protocols and recommendations provided by the Puerto Rico MTF COVID-19 were formulated in accordance with evidence-based public health principles on how to handle a pandemic. The working groups were defined using the WHO priority areas of work: emergency response, hospital response and management, laboratory tests for COVID-19, surveillance and contact tracing, risk communication and community engagement, infection prevention and control, and societal response [4]. Table 1 presents the recommendations given by the MTF on each of these areas, which included suggestions on which government agencies would be in charge of the implementation of the proposed public health responses.

During the first three weeks since its inception, these working groups met on an almost daily basis to revise the scientific literature and national and international guidelines related to their topic prior to preparing culturally adapted protocols on how to apply the scientific evidence to Puerto Rico. The principle guiding the development of the protocols was that COVID-19 will need a comprehensive public health response that included all members of the Puerto Rican society. The response to COVID-19 was spearheaded by the Puerto Rico Department of Health and implemented by various entities such as the Puerto Rico State Agency for Emergency and Disaster Management, private and public health services, manufacturing companies, and many others.

In terms of the specific public health response, the Puerto Rico MTF COVID-19 was established under the understanding that this was a new disease, comparable but significantly different to other viral infections, such as influenza, with many unknowns in regards to its methods of transmission and the risk factors for severe disease. The members of the MTF also considered that the COVID-19 pandemic evolved dynamically and generated protocols and recommendations that needed constant revisions to reflect advancements in scientific knowledge.

### 2.3. Measures of Impact and Projections

One of the MTF’s key roles was to estimate the future needs for the Puerto Rican healthcare system in response to the COVID-19 epidemic. To accomplish this goal, the literature and the behavior of the COVID-19 infection was reviewed, and thus it was estimated that there were many more people infected with COVID-19 than those detected by the available laboratory tests. This discrepancy could be attributed to the high number of asymptomatic individuals infected with COVID-19 and the limited testing capacity for COVID-19 during the epidemic.

An alternative way to estimate the course of the epidemic is to back-calculate infections from observed deaths. Reported deaths are likely to be more a more reliable method to estimate infections [5]. Hence, we developed estimates for future healthcare resources needed, including hospital intensive care unit (ICU) beds and ventilators using a fatality-based model [5,6]. The model takes into account a time lag in observing the effect of interventions on deaths since there is a 2–3-week period between infection, onset of symptoms, and outcome. Our methods assumed that changes in infection transmission are an immediate response to the government interventions being implemented (such as physical distancing and lockdown measures) rather than broader gradual changes in behavior. Our model thus estimated changes in community COVID-19 transmission by calculating backward from the deaths observed over time to estimate transmission that occurred several weeks prior, allowing for the time lag between infection and death. The expected number of deaths on a given day is a function of the number of infections occurring in previous days, thus adjusting daily increase in infections to match percent changes in deaths. To measure outcomes and the impact of our recommendations, we also compared transmission and number of cases in Puerto Rico with reported cases in other geographic areas in the U.S.

### 2.4. Statistical Methods

The cumulative number of confirmed and probable cases, by date of sample collection, was described based on the number of specimens tested for SARS-CoV-2 and reported to the Puerto Rico Department of Health public health laboratory as well as those reported by local clinical and commercial laboratories. Similarly, cumulative confirmed and probable deaths were described by date of event. All deaths occurring among persons with laboratory-confirmed SARS-CoV-2 infection were matched to the death certificates provided by the Puerto Rico Department of Health Demographic Registry. Those deaths without laboratory confirmation for which COVID-19, SARS-CoV-2, or an equivalent term was listed on the death certificate as an immediate, underlying, or contributing cause of death were classified as probable deaths.

To predict the cumulative number of COVID-19 cases in Puerto Rico, a nonlinear regression model using the Gompertz growth function was used as follows:

$$\mu_{i}=\beta_{1}*e^{-\beta_{2}*e^{\left(-\beta_{3}t\right)}}$$

where *μ_i_* is the cumulative number of confirmed cases, *β*_1_ is the predicted maximum number of confirmed cases, *β*_2_ and *β*_3_ are fitting coefficients, and t is the number of days since the first case [7,8,9]. The nonlinear least-squares estimation command with the Gompertz function in Stata version 16 (StataCorp LLC, College Station, Texas, USA, 2019) was used to predict the number of cases.

### 2.5. Healthcare Utilization Estimation Analysis

The following assumptions were made based on published literature estimating the behavior of the disease from exposure to hospitalization and death [10,11]. We estimated the mean duration from onset of symptoms to death to be 14 days (95% CI: 16.9–19.2) and a 7-day incubation period from exposure to COVID-19 testing. Using the age-distribution of the population of Puerto Rico from the Census data (3.2 million people), we obtained an estimate of the expected number of infections and fatality rates in each age group based on global cohorts data [5,6]. We assumed an attack rate of 10% for the Puerto Rican population, where the maximum total number of people exposed would be 30,698. The probability of becoming infected was equal across all age strata. We linked our function of deaths to infected cases, using a previously estimated COVID-19 infection–fatality ratio (probability of death given infection) together with a distribution of times from infection to death. Model fitting for ICU utilization at 30 days was done by the Gompertz model fitted with a lower limit at 0 (3 parameters). Analysis was done with R software version 3.6.2 (R Core Team, Vienna, Austria, 2019).

The Institutional Review Board (IRB) recognizes that the analysis of de-identified, publicly available data does not constitute human subjects research as defined at 45 Code of Federal Regulations (CFR) 46.102, and therefore, it does not require IRB review.

## 3. Results

**Figure 1:**
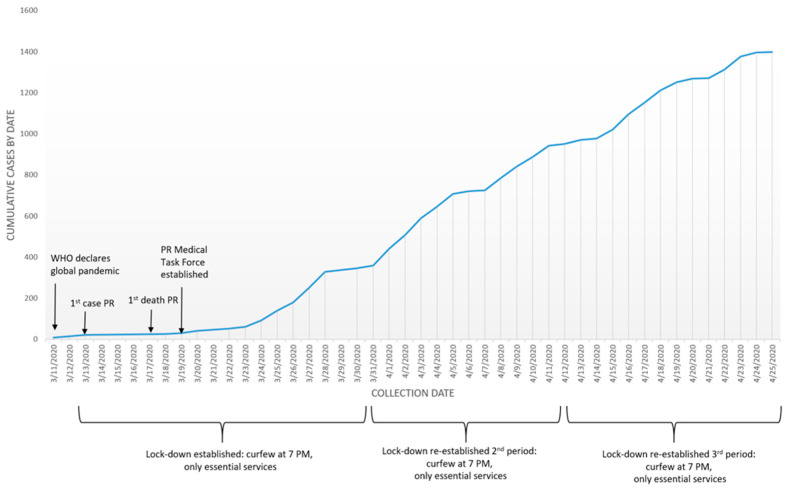
Public health response to the coronavirus disease 2019 (COVID-19) pandemic in Puerto Rico.

One of the first tasks of the Puerto Rico MTF was to assess the public health system’s readiness to cope with the epidemic. To complete this assessment, we evaluated the ability to rapidly test for the infection, the coverage of tracking and isolating cases and their contacts, and the medical capacity to manage the surge in COVID-19 cases. As of June 2020, there are still uncertainties regarding the accuracy of surveillance reports of cases, the effectiveness of contact tracing to prevent the continued spread of the illness, and the number of tests performed in Puerto Rico, indicating the possibility that such efforts were not enough. As an advisory board, the MTF provided evidence-based recommendations, but the Government of Puerto Rico did not implement all of our guidelines. The MTF worked collaboratively with the Government of Puerto Rico to advise on the public health response to this pandemic (Figure 1). From the MTF recommendations, the principal activities that the Government implemented were early lockdown/shelter-in-place, universal mask-wearing, and physical distancing measures.

### 3.1. To Provide Clinical Guidelines on the Appropriate Medical Management of Critically Ill and Non-Critically Ill Patients

One of the main tasks of this scientific collaboration was to provide guidelines to support the implementation of response activities at different levels of impact. The guidelines developed targeted not just clinical management, but other critical areas for the response, including surveillance and contact tracing, risk communication, healthcare regulatory issues, and societal response. Table 1 provides a summary of the committees, protocols, and recommendations created and agencies that acted during each specific area’s implementation phase.

### 3.2. To Reduce the Transmission of the Virus by Doing Rapid Detection of Cases That Would Prevent Deaths

**Figure 2:**
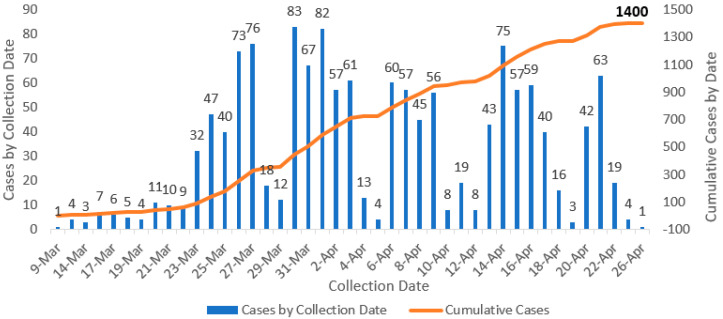
Cumulative number of confirmed and probable cases by sample collection date in Puerto Rico, as of 26 April 2020.

The epidemiological data demonstrated a decrease in the growth rate of the epidemic after the implementation of the shelter in place order and the establishment of the MTF (Figure 2).

**Figure 3:**
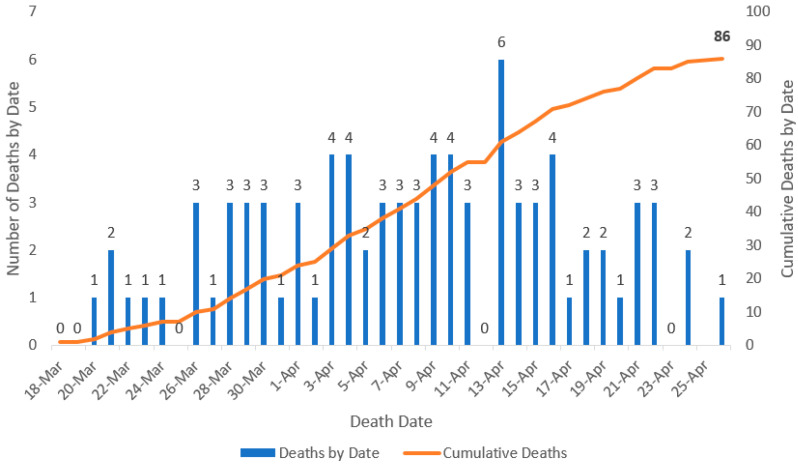
Cumulative number of laboratory-confirmed and probable COVID-19 deaths in Puerto Rico, as of 26 April 2020.

After two weeks of establishing the epidemic response in Puerto Rico, the daily growth curve of positive results in COVID-19 testing decreased from 27.1% to 11.5%. There was also a decrease in the number of deaths after 15 April 2020 (Figure 3).

**Figure 4:**
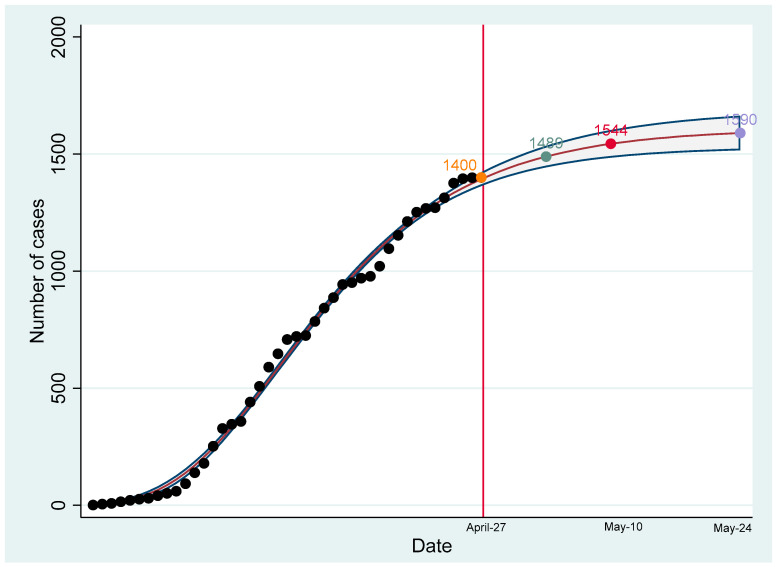
Daily confirmed COVID-19 cases in Puerto Rico as of 27 April 2020 and predictions according to Gompertz model.

The projection of the cumulative number of confirmed and probable cases showed a flattening of the growth curve by 22 April 2020 (Figure 4). As of 23 June 2020, data reported by the United States Centers for Disease Control and Prevention (U.S. CDC) indicate that the cumulative incidence rate of COVID-19 per 100,000 population in Puerto Rico was 209.2 [12]. States with similar populations, such as Iowa and Nevada, have a much higher cumulative incidence rate per 100,000 population (825.4 and 453.6, respectively) [12]. Non-contiguous states, such as Hawaii and Alaska, have significantly much lower rates (54.3 and 103.2, respectively) [12]. On the other hand, Mississippi (746.3) and Arkansas (533.6), states with similar poverty levels as Puerto Rico [13], had significantly higher rates. However, West Virginia, another state with a similar poverty level had a lower rate (142.4) [12]. Notably, the Governor of West Virginia named an academic (Dr. Clay Marsh, Vice-President and Executive Dean for Health Sciences at West Virginia University) as the state’s COVID-19/Coronavirus Czar and issued a state-at-home order on a similar date (23 March 2020) to Puerto Rico [14].

**Figure 5:**
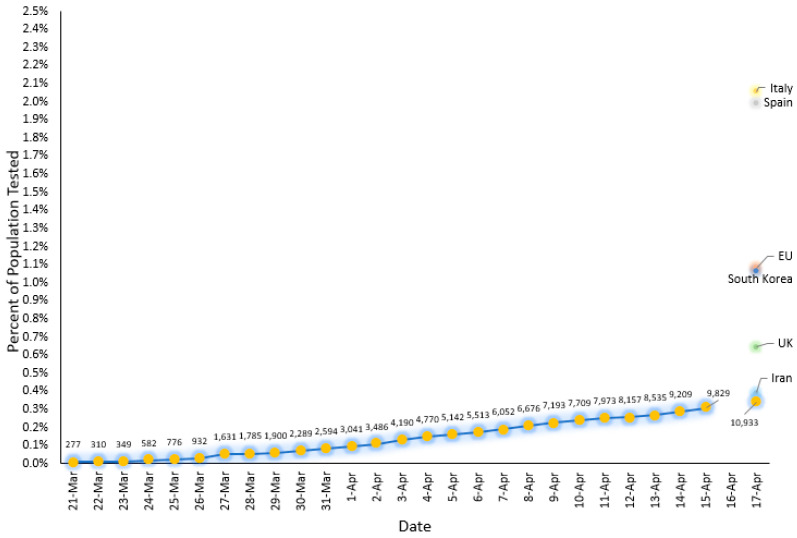
Diagnostic testing capacity as measured by percent of the population tested in Puerto Rico by sample collection date. Data points from other countries are included for reference (Source: data from the Puerto Rico Department of Health and *The New York Times* gathered and analyzed by Drs. Linnette Rodríguez and Héctor Colón).

However, the reduced diagnostic capacity for testing COVID-19 in Puerto Rico limited our projections (Figure 5).

Although comparison of COVID-19 diagnostic capacity across states and countries is difficult due to variations in economic, political, and social factors, including the self-sufficiency to obtain the diagnostic tests and the healthcare system’s capacity to administer them, Puerto Rico fell behind in the number of tests performed (less than 0.2% of the population) [15]. As of 25 June 2020, the total tests per million people in Puerto Rico were only 3845 [16]. The U.S. state closest in the number of tests to Puerto Rico is Idaho, with more than ten times (44,800) tests per million completed [16]. Even if we compare testing done in non-contiguous states such as Hawaii (58,834 tests per million) and Alaska (135,948 tests per million), there continues to be a marked disparity in the number of tests performed in Puerto Rico [16]. The only two other territories of the U.S. with a lower number of tests are Guam (10,833 tests performed in a population of 165,768) and the U.S. Virgin Islands (2755 in a population of 106,977) [16]. It is essential to state that these comparisons are difficult to make given that test data reporting is not uniform across states and territories. In addition, we acknowledge the limitation that even as of May 2020, there are inconsistent reports on the number of rapid serological tests versus molecular diagnostic tests used for reported cases.

### 3.3. To Minimize the Impact the Epidemic Could Have on the Health System, Social Services, and the Community

**Figure 6:**
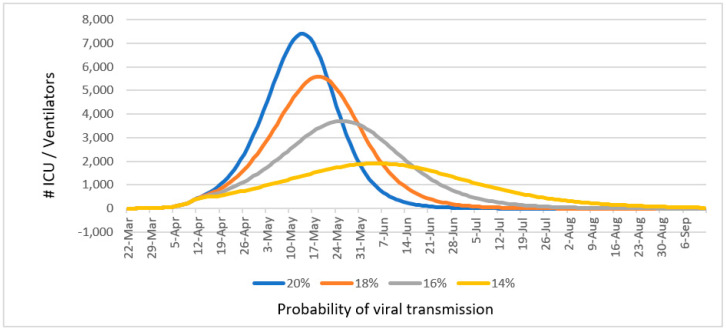
Projected intensive care unit (ICU) and mechanical ventilator needs based on different probabilities of viral transmission (14%, 16%, 18%, and 20%).

To estimate health services utilization focused on ICU and mechanical ventilation needs, our group used a fatality-driven model using data on COVID-19 infection behavior as described in the methods section. In addition, observed fatality rates of 35–40% among ICU admissions and 66–75% for those who require mechanical ventilation were incorporated to increase the robustness of the model [17]. The ICU/ventilator estimated capacity for the island to manage the COVID-19 pandemic at various infection rates is presented in Figure 6. These estimates guided healthcare system preparedness strategies, including the purchase of equipment by the Puerto Rico Department of Health.

## 4. Discussion

The Puerto Rico MTF COVID-19 was a volunteer academic-based entity formed to assist the government in the response to the public health crisis of COVID-19. The use of this model for the rapid response to a pandemic appears to have been effective for Puerto Rico, as determined by the flattening of the curve of infection within a period of five weeks from initiation of the government-imposed lockdown measures. This allowed for healthcare system and economic system preparedness resulting in lives saved and a coordinated integration of services. The epidemiological data have shown that this academic and government collaboration has been able to slow the growth of cases with a projected flattening of the growth curve by 22 April 2020. This has been done even in the face of a low availability of molecular COVID-19 tests in relation to the baseline population. This highlights that in a small island territory such as Puerto Rico with limited access to COVID-19 tests, implementation of shelter-in-place, social/physical distancing, and academic–government collaboration were essential first steps in a pandemic response. It seems that a similar alliance between government and academia also worked for at least one U.S. state, West Virginia.

In terms of general recommendations for the prevention of disease dissemination and to reduce and stop the transmission of the virus, the Puerto Rico MTF COVID-19 recommended the use of social-physical distancing techniques and provided guidance to the Governor on how to use the available data to develop the guidelines on how to implement social-physical distancing. We also recommended the use of personal protection equipment (PPE) depending on the level of exposure and based on the U.S. CDC guidelines and literature available at the time. This included the use of a facemask or a cloth face covering for all citizens in public settings. Universal face covering was implemented and reinforced since 31 March 2020, several days prior to the WHO and the U.S. CDC equivalent statements on this matter [18,19]. Mandated face covering reduces the number of infections [20] and we believe this protective measure has contributed to keep the pandemic under control in Puerto Rico.

The lockdown in Puerto Rico extended from 15 March to 15 June 2020. During this period, there was also a curfew (from 7:00 p.m. to 5:00 a.m.). To this date, the lockdown has been suspended, but a modified curfew (from 10:00 p.m. to 5:00 a.m.) is still in place. Although this is a rather prolonged lockdown period as compared to other regions, transmission rate has remained stable in Puerto Rico even after the reopening of economic activities. This finding is congruent with the data from López and Rodó, who using a model to explore different post-confinement scenarios concluded that “lockdowns should remain in place for at least 60 days to prevent epidemic growth” [21].

The MTF also provided recommendations for all elective or non-emergency clinical services to be suspended or offered through the rapid implementation of telehealth services. These recommendations included that all elective surgeries were suspended. Only emergency surgeries, Tier 3a and 3b categories under the American College of Surgeons Surgical Guidelines [22], were allowed to be performed during this period. One of the most urgent requests made by the MTF was also to recommend that all air transportation into the island was to be channeled through only one airport, the Luis Muñoz Marín International Airport. This permitted setting up airport surveillance guidelines that included that all incoming passengers who had any symptoms were recommended to be tested with a PCR or rapid serological test for COVID-19. Thermal imaging monitors were also set up in the airport and recommendations were provided on how to handle passengers who presented with fever, including testing them as well as performing contact tracing of all passengers who were seated nearby on the plane. Members of the Puerto Rico National Guard and volunteer medical students from the UPR and other medical schools on the island collaborated in this operation in the airport.

Another important contribution was the recommendation on how to implement the evidence-based guidelines on screening for the disease in Puerto Rico. The principal screening mechanism was funneled through primary care and specialty physicians in the community. The MTF emphasized the use of telehealth or telemedicine methods to screen for COVID-19 and used community health services for testing using the criteria established by the U.S. CDC. The MTF also provided recommendations on how to increase the testing capacity in Puerto Rico, including using university or private laboratories that could obtain the needed certifications to test. It also provided recommendations on how to improve the reporting of positive tests and how to communicate the testing data to the community. In addition, the task force offered recommendations and worked hand in hand with the Epidemiology Division of the Department of Health for contact tracing and dissemination of the data.

The MTF provided support to primary and specialty care physicians on the appropriate medical management of cases. It created protocols on treatment guidelines on how to treat outpatient cases through home isolation as well as reviewed all the literature on observational data of possible treatments for more severe inpatient cases. The MTF also advocated for legislation to restrict the use of needed medications, such as hydroxychloroquine for hospital cases, adherence to changing guidelines on the prescribing of controlled substances, handling of medication prescribing through online methods, and offering of pharmaceutical services by community pharmacies incorporating measures conducive to social distancing. It also provided recommendations on how to distribute PPE to health professionals. Other important contributions of the Puerto Rico MTF COVID-19 included guidelines on how to handle deaths. The MTF provided recommendations on how to diagnose COVID-19 in people who had died before completing the test, how to manage loss and grief in families of the deceased, and how to handle corpses. The group was also very receptive in terms of addressing vulnerable and at-risk populations. Therefore, specific guidance to nursing and convalescent homes was developed including all at-risk staff personnel and first responders including hospital personnel, paramedics, police, firefighters, etc.

The evidence on how to improve treatment for COVID-19 included assessing ways to increase the health system’s capacity in order to minimize the impact the epidemic could have on Puerto Rico’s healthcare system and the community at large. From this work, the Puerto Rico MTF COVID-19 recommended fast-track credential evaluations for fourth year medical students, graduating nursing students, and retired health professionals. The MTF also recommended the creation of medical hubs outside of traditional health services such as using hotels or convention/concert venues. In view of lack of sufficient tests and disparity in testing capacity in Puerto Rico, as compared with other regions, the MTF used a fatality-based model to estimate hospital, ICU, and ventilator utilization. This model provided an adequate estimation of healthcare use and could be used in other regions with gaps in testing capacity.

The MTF also met with other government-appointed working groups, such as the Economic Task Force and the Social Task Force, to ensure that all medical recommendations took into consideration the economic and social effects on the Puerto Rican community. Our collaboration with the Economic Task Force is expected to continue until after the stepwise economic reopening occurs to provide scientific data during the decision-making process in an attempt to guarantee the safety of employees and the public and to prevent a collapse in the healthcare system.

## 5. Conclusions

The incorporation of a national MTF with members of the academia to leverage government resources was beneficial during the initial COVID-19 response in Puerto Rico. A similar strategy could serve as a model for other states or territories and countries in similar scenarios for a rapid and orchestrated response to prepare, educate, and save lives. However, we should continue to increase testing capacity, improve surveillance (especially contact tracing), reinforce physical distancing, and encourage other preventive measures to strengthen the Puerto Rican healthcare system while educating the community and promoting overall physical and mental well-being.

## Figures and Tables

**Table 1:** Puerto Rico Medical Task Force (MTF) COVID-19 working group committees, protocols created, and affiliated implementation agencies.

Committee	Protocols and Recommendations	Implementation Agencies
Emergency Response	Recommendation for a coordinated response to the COVID-19 epidemic in Puerto Rico Recommendation for centralized purchasing and distribution of emergency-related supplies	Puerto Rico Emergency Management and Disaster Administration Department Puerto Rico Department of Health Puerto Rico National Guard
Hospital Response and Management	Increase availability of hospital beds and equipment, including intensive care unit (ICU) beds and ventilators Increase clinical staff available by expediting credentialization Increase the use of telehealth Clinical management of COVID-19 protocols (home isolation, treatment guidelines for hospitalized patients, use of plasma from recovered patients, management of mental health conditions, discontinuation of home isolation) Protocol for the management of corpses	Puerto Rico Department of Health Federal Emergency Management Agency Puerto Rico Office of Certification of Health Professionals Health Professionals’ Associations Public and private health services institutions Forensic Sciences
Laboratory Tests for COVID-19	Protocols for the assessment of COVID-19 in adults, pediatric, and pregnant populations Protocol for the assessment of COVID-19 among passengers in airports Recommendations for expanding testing capacity	Puerto Rico College of Medical Technologists Clinical laboratories Scientific community
Contact Tracing	Recommendations for reporting and following confirmed cases Recommendations for identifying and following contacts Protocol for the isolation of suspected and confirmed cases of COVID-19 Protocol for the domiciliary quarantine of contacts of suspected and confirmed cases of COVID-19 Protocol for the quarantine of suspected and confirmed travelers detected at the airport Protocol for screening first responders	Puerto Rico Department of Health Public and private hospitals Clinical laboratories
Surveillance	Recommendations for rapid surveillance of COVID-19 in Puerto Rico Protocols for surveillance of suspected and confirmed cases in hospitals, laboratories, and other health services’ facilities Protocol to report confirmed and suspected deaths	Puerto Rico Department of Health Public and private hospitals Clinical laboratories
Risk Communication and Community Engagement	Recommendations for disseminating data to the community Dissemination of the clinical relevance of epidemiological data to the community Development and dissemination of prediction models with the community	Puerto Rico Department of Health Government Communication Offices Puerto Rico Statistics Institute
Infection Prevention and Control	Protocol for the use of personal protective equipment Protocols for infection prevention in special populations and essential services	Puerto Rico Department of Health Puerto Rico National Guard Correctional System Extended care facilities Public and private hospitals Professional organizations Mayors of municipalities of Puerto Rico Commercial sector
Societal Response	Recommendations for a sustainable recovery from COVID-19 Recommendations for meeting social needs	Puerto Rico Economic Task Force COVID-19 Puerto Rico Social Task Force COVID-19
